# Ribosomal transcription is regulated by PGC-1alpha and disturbed in Huntington’s disease

**DOI:** 10.1038/s41598-017-09148-7

**Published:** 2017-08-17

**Authors:** Sarah Jesse, Hanna Bayer, Marius C. Alupei, Martina Zügel, Medhanie Mulaw, Francesca Tuorto, Silke Malmsheimer, Karmveer Singh, Jürgen Steinacker, Uwe Schumann, Albert C. Ludolph, Karin Scharffetter-Kochanek, Anke Witting, Patrick Weydt, Sebastian Iben

**Affiliations:** 1 Department of Neurology, University of Ulm, Oberer Eselsberg 45, 89081 Ulm, Germany; 2 Experimental Neurology, University of Ulm, Helmholtzstrasse 8/1, 89081 Ulm, Germany; 3 Clinic of Dermatology, University of Ulm, James-Franck Ring N27, 89081 Ulm, Germany; 4 Department of Internal Medicine II, University of Ulm, Parkstrasse 11, 89075 Ulm, Germany; 5 Institute for Experimental Cancer Research, University of Ulm, James-Franck Ring N27, 89081 Ulm, Germany; 6 Division of Epigenetics, DKFZ-ZMBH Alliance, German Cancer Research Center, INF 580, 69120 Heidelberg, Germany

## Abstract

PGC-1α is a versatile inducer of mitochondrial biogenesis and responsive to the changing energy demands of the cell. As mitochondrial ATP production requires proteins that derive from translation products of cytosolic ribosomes, we asked whether PGC-1α directly takes part in ribosomal biogenesis. Here, we show that a fraction of cellular PGC-1α localizes to the nucleolus, the site of ribosomal transcription by RNA polymerase I. Upon activation PGC-1α associates with the ribosomal DNA and boosts recruitment of RNA polymerase I and UBF to the rDNA promoter. This induces RNA polymerase I transcription under different stress conditions in cell culture and mouse models as well as in healthy humans and is impaired already in early stages of human Huntington’s disease. This novel molecular link between ribosomal and mitochondrial biogenesis helps to explain sarcopenia and cachexia in diseases of neurodegenerative origin.

## Introduction

The main source of cellular ATP in eukaryotes is oxidative metabolism in the mitochondria and the activity and amount of mitochondria is subject to regulation by a transcriptional network. A key transcriptional regulator of mitochondrial biogenesis is the peroxisome proliferator-activated receptor gamma co-activator 1α (PGC-1α)^1^. PGC-1α is detectable in tissues with high energy demand such as heart, skeletal muscle, brown adipose tissue (BAT), liver and brain^2^. Beside mitochondrial biogenesis PGC-1α regulates different metabolic adaptation processes like gluconeogenesis in the liver^3^, glucose uptake and fatty acid oxidation in the skeletal muscle^4^, adaptive thermogenesis in BAT^5^, fatty acid oxidation in heart^6^ and neuronal energy homeostasis and ROS-detoxification in the brain^7, 8^. Dysregulation of PGC-1α has been connected to many neurodegenerative and metabolic disorders; Parkinson’s, Alzheimer’s and Huntington’s disease, Amyotrophic Lateral Sclerosis (ALS)^9, 10^, type 2 diabetes^11^, obesity^12^ and heart failure^13, 14^ are just some examples. PGC-1α itself is subject to intensive regulation on transcriptional and posttranslational level, thus the amount of PGC-1α expression, the subcellular localization and its phosphorylation, acetylation and sumoylation state control its activity. PGC-1α is a master regulator of metabolic adaptation and responds to diverse stressors. The activity of PGC-1α is modulated by fasting or the calorie-restriction mimetic resveratrol^15^, by altered oxygenation^14^ or ß-adrenergic stimulation^16^ to name a few. Knock-out of PGC-1α is viable, but the animals display failures in thermogenesis and energy homeostasis and are lean and hyperactive due to striatal degeneration patterns reminiscent of the neurodegenerative disorder Huntington’s disease^17^. In fact it has been shown, that mutant huntingtin negatively impacts the expression of PGC-1α^10, 18^.

Ribosomes are responsible for the synthesis of all cellular proteins. The key step in ribosomal biogenesis is transcription of the rDNA by RNA polymerase I and its specific transcription factors in the nucleolus, where the pre-ribosomes are assembled.

Mitochondria possess mito-ribosomes that in humans translate 13 different proteins of the respiratory chain. The majority of mitochondrial structure and biogenesis is dependent on the translation products of the cytosolic ribosomes^19^, thus mitochondrial biogenesis is at least partially dependent on ribosomal biogenesis. Therefore, we speculated that ribosomal biogenesis and mitochondrial biogenesis might be linked by a common transcriptional regulator like PGC-1α. Here, we demonstrate that PGC-1α locates to the nucleolus, associates with the rDNA and controls rDNA transcription in response to multiple stimuli in several tissues and cell types. Moreover, we identify PGC-1α as a tissue specific modulator of rDNA transcription, a function that is impaired in individuals carrying the Huntington’s disease mutation.

## Results

### PGC-1α localizes to the nucleolus and associates with the unmethylated rDNA

**Figure 1:**
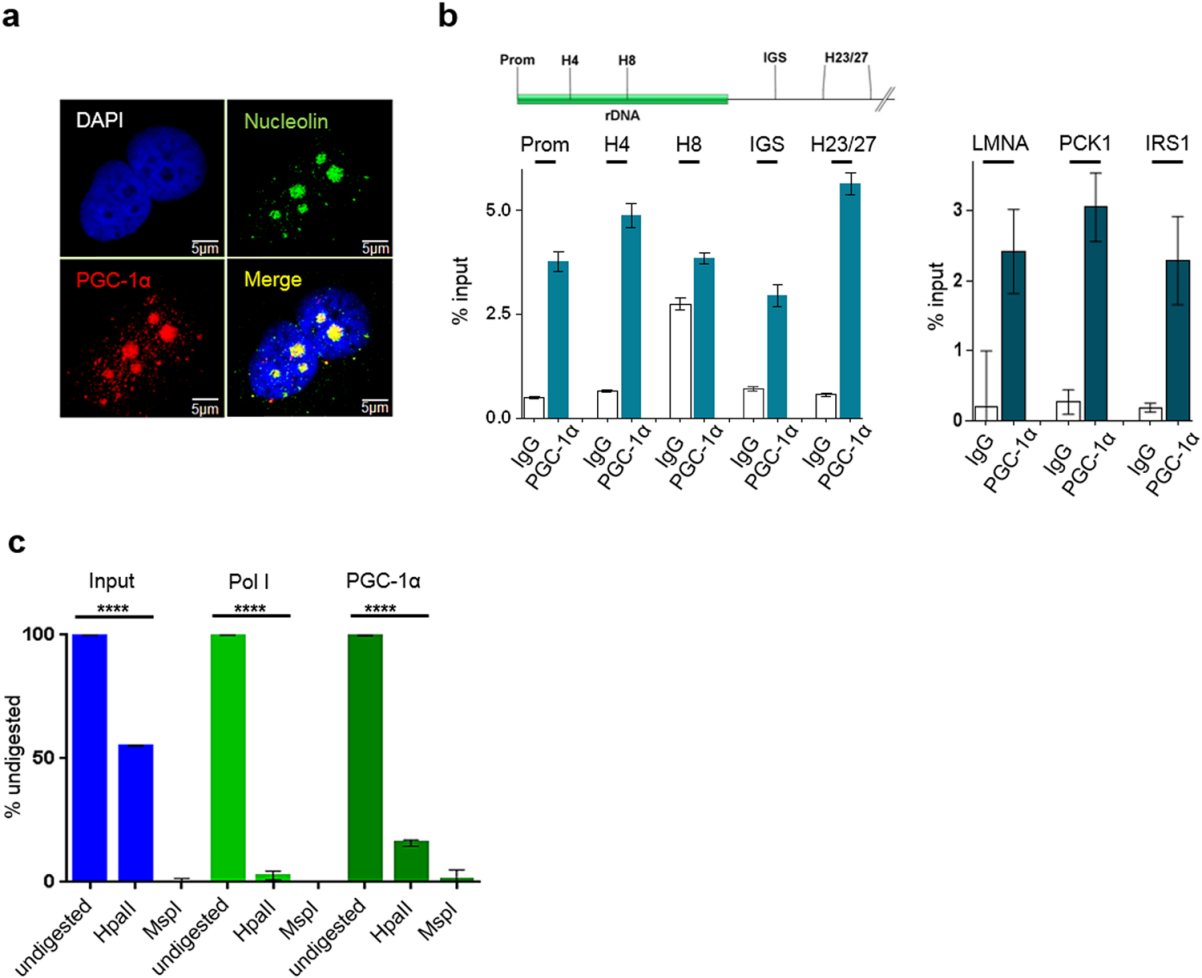
Localization of PGC-1α in the nucleoli and association with the unmethylated rDNA. (**1a**) PGC-1α is condensed located in the nucleolus as shown by the merge with nucleolin (staining of the nucleoli). Confocal microscopy of untreated HEK cells kept under 21% pO_2_. N = 155 nucleoli of HEK cells were investigated for statistical analysis (Figure S2). (**1b)** PGC-1α associates with the rDNA-promotor (Prom), gene internal sequences (H4, H8) and the intergenic spacer (IGS, H23/27). QPCR of ChIP assay with HEK cells and PGC-1α antibody (Calbiochem). Samples were normalized to input (native chromatin as positive control). N = 3; one way ANOVA; column: mean ± SD. Control experiments using bona fide PGC-1α targets like LMNA (lamin A), PCK1 (phosphoenolpyruvate carboxykinase 1) and IRS1 (insulin receptor substance-1) show a clear association of PGC-1α with these genes. qPCR of ChIP experiments, n = 3, column: mean ± SD. (**1c**) PGC-1α associates with the unmethylated, active rDNA-promotor. ChIP assay with HEK cells kept under 21% pO_2_, transfected with flag-tagged PGC-1α. Digest with methylation-sensitive enzymes Hpa II (digests only unmethylated sequences) and Msp I (digests both methylated and unmethylated sequences). qPCR with HrChIP-primer. Pol I and PGC-1α were normalized to the undigested chromatin. ****p < 0.0001; one way ANOVA; N = 6; column: mean ± SD.

The transcriptional co-activator PGC-1α shuttles between cytosol and nucleus depending on its activation state^20^. Asking if the nuclear PGC-1α co-localizes to the site of rDNA transcription, the nucleolus, we performed immunocytochemical staining under ambient O_2_ concentrations in PGC-1α expressing HEK cells. Confocal microscopy revealed a clear enrichment of PGC-1α in nucleoli (Fig. 1a). This nucleolar localization was confirmed in N2A cells (Supplementary Figure S1). Quantification of confocal microscopy revealed a significant overlap between mean signal intensities of PGC-1α and the nucleolar marker nucleolin in both cell lines (Supplementary Figure S2). To investigate PGC-1α localization under near physiological conditions, we repeated the experiment at 3% O_2_ and found predominant localization of the transcriptional cofactor in the cytoplasm. When activated by addition of resveratrol, PGC-1α again shuttles to the nucleolus (Supplementary Figure S3a). The expression level of PGC-1a does not change between 3% and 21% O_2_ neither on mRNA nor on protein level (Supplementary Figure S3b).

Next we were interested if PGC-1α associates with the rDNA. We performed chromatin-immunoprecipitation experiments with HEK cells grown under ambient O_2_ concentrations. PGC-1α antibodies precipitated promoter and gene-internal sequences of the rDNA and sequences of the non-coding intergenic spacer including a duplicate sequence 23 and 27kb downstream of the promoter (Fig. 1b, Supplementary Figure S4). To validate the ChIP results, we analysed the co-precipitation of *bona fide* PGC-1α targets and found enrichment of promoter fragments of lamin A, phosphoenolpyruvate carboxykinase 1 and insulin receptor substrate-1 (Fig. 1b). 50% of the rDNA copies of a cell are inactivated by promoter methylation, thus the methylation pattern of a ChIPed rDNA promoter was analyzed by differential digestion via methylation sensitive isoschizomeric enzymes^21^. Fig. 1c shows that almost 90% of the rDNA promoter ChIPed by PGC-1α antibodies is sensitive to HpaII that digests only unmethylated sequences, indicating that PGC-1α associates preferentially with the active fraction of rDNA promoters.

### Activated PGC-1α associates with rDNA, stimulates promoter occupancy and interacts with the upstream binding factor (UBF)

**Figure 2:**
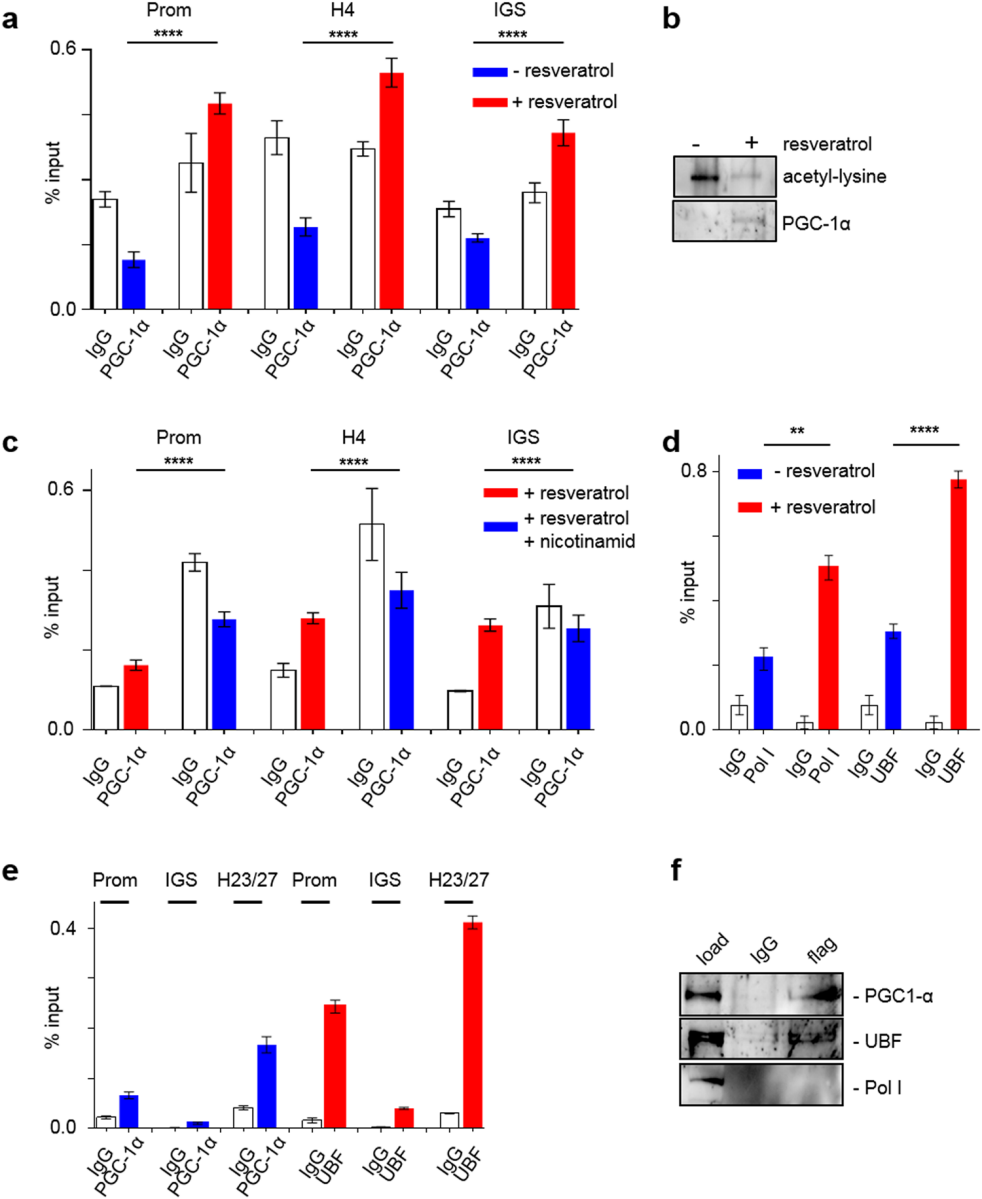
The rDNA association of PGC-1α is dependent on its activation state and influences promoter occupancy of the initiation complex. (**2a**) PGC-1α associates with the rDNA-promotor, gene internal sequences and the intergenic spacer only after stimulation with resveratrol. ChIP assay with untransfected HEK cells, kept under 3% pO_2_, treated with/without resveratrol (10 µM) for 3 h. Samples were normalized to input (native chromatin, positive control) N = 3; ****p < 0.0001; column: mean ± SD. (**2b**) Resveratrol treatment induces deacetylation of PGC-1α. Immunoprecipitation of lysates of flag-PGC-1α transfected cells treated without/with 10 µM resveratrol with anti-flag (M2) antibodies. The membrane was probed with acetyl-lysine antibody and, after stripping, with anti-flag antibody to detect flag PGC-1α. (**2c**) The effect of resveratrol on PGC-1α-association with the rDNA can completely be reversed by addition of the resveratrol antagonist nicotinamide (10 mM) for 3 h. ChIP assay. Samples were normalized to input (native chromatin, positive control). N = 3; ****p < 0.0001; column: mean ± SD. (**2d**) Promotor occupancy of Pol I and UBF is increased in HEK cells, treated with 10 µM resveratrol for 3h. HEK cells kept under 3% pO_2_ with/without resveratrol; ChIP assay, qPCR using promoter primer. Samples were normalized to input (native chromatin, positive control). N = 3; **p = 0.0022, ****p < 0.0001; column: mean ± SD. (**2e**) PGC-1α and UBF associate with the same rDNA sequences. ChIP-re-ChIP. Samples were normalized to input (native chromatin, positive control). N = 3, mean ± SD. (**2f**) Co-immunoprecipitation of PGC-1α with subsequent western blot analysis using UBF and RNA polymerase I antibodies (RPA135). Nuclear extracts of flag-tagged PGC-1α transfected HEK cells. N = 3. Anti-Flag ab for detection of PGC-1α 1:500. (Full-length blot see supplement)

Next we investigated whether association of PGC-1α with rDNA is also dependent on environmental conditions. We prepared chromatin from untransfected HEK cells cultivated under 3% O_2_ in the absence/presence of resveratrol. Subsequent ChIP-analysis revealed that PGC-1α only associates with rDNA when activated by resveratrol (Fig. 2a). Resveratrol induced de-acetylation of transfected PGC-1α at 3% Ο_2_ as analysed by immunoprecipitation and probing with acetyl-lysine antibody (Fig. 2b). The association of PGC-1α with the rDNA promoter was prevented by addition of nicotinamide (Fig. 2b), a resveratrol antagonist^15^.

To elucidate the effects of PGC-1α association with the rDNA on the transcription initiation complex, we immunoprecipitated Pol I and UBF of HEK cells and investigated whether PGC-1α association with the rDNA correlates with promoter occupancy of Pol I and UBF. Addition of resveratrol to HEK cells significantly increased the amount of rDNA promoters immunoprecipitated with Pol I and UBF antibodies (Fig. 2c). To further investigate a possible interaction of PGC-1α with UBF, we performed ChIP-re-ChIP experiments from chromatin at ambient O_2_. As depicted in Fig. 2d, UBF antibodies immunoprecipitated the same promoter and intergenic spacer sequences as PGC-1α antibodies indicating that both proteins interact with the same molecules of rDNA. In control experiments we find a strong binding of UBF to the non-coding regions amplified by H23/27 (Supplementary Figure S4). Co-IP experiments with nuclear extracts of HEK cells revealed that PGC-1α interacts with significant amounts of UBF, but not RNA polymerase I, indicating that PGC-1α might serve as a co-factor of UBF (Fig. 2e).

### PGC-1α stimulates rDNA transcription

**Figure 3:**
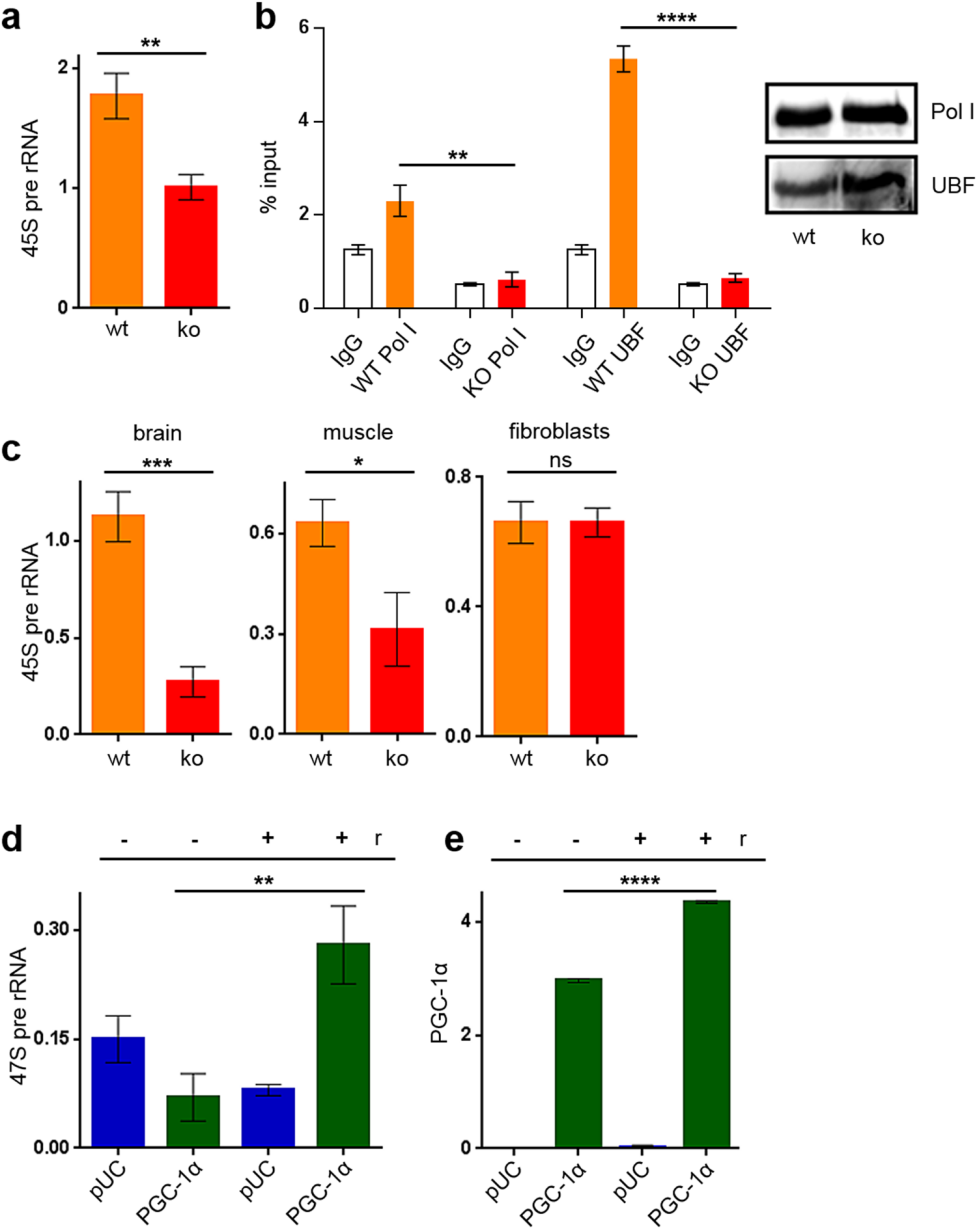
PGC-1α stimulates rDNA transcription *in vitro* and *in vivo*. (**3a**) PGC-1α-KO tissue (BAT) show reduced rDNA transcription. QPCR with m45S-primer from wt (orange) and ko (red) BAT cDNA. N = 3, **p = 0.0037; one way ANOVA; column: mean ± SD. (**3b**) Promoter occupancy of the initiation complex in KO tissue in comparison to WT. QPCR and Western blots of ChIPs from wt (orange) and ko (red) BAT chromatin N = 3, **p = 0.0013; ****p < 0.0001; one way ANOVA; column: mean ± SD. (Full-length blots see supplement). (**3c**) RNA polymerase I transcription analysis of WT and PGC-1α-KO tissues and cultivated mouse fibroblasts. qPCR with m45S-primer. N = 3; *p = 0.0134, ***p = 0.0006; one way ANOVA; column: mean ± SD. (**3d**) Treatment with 10 µM resveratrol for 3h increases transcription of the 47 S rRNA in cell culture. HEK-cells kept under 3% pO_2_, transfected with flag-tagged PGC-1α and pUC18. The panels are a representative of four independent experiments; **p = 0.0044, one way ANOVA; column: mean ± SD. (**3e**) PGC-1α-expression after 3 h treatment with resveratrol (10 µM). HEK-cells kept under 3% pO2, transfected with flag-tagged PGC-1α and pUC18. ****p < 0.0001; one way ANOVA; column: mean ± SD.

To investigate the functional relevance of PGC-1α association with the rDNA, we compared the initiation rate of RNA polymerase I transcription using primers recognising the 5’part of the 45S pre-rRNA in tissues of PGC-1α wild-type and knock-out animals. The 45S pre-rRNA precursor is posttranscriptionally cleaved and an indicator of ongoing transcription at the time point of harvest. BAT from PGC-1α knock-out animals displayed lower RNA polymerase I transcription than tissue from wild-type mice (Fig. 3a). Moreover, when promoter occupancy of Pol I and UBF was compared between PGC-1α competent wild-type and PGC-1α knock-out tissue, we found a highly significant difference of Pol I and UBF binding to the rDNA promoter after precipitating the same amount of protein (Fig. 3b).

Reduced RNA polymerase I transcription was also detected in brain and muscle of PGC-1α knock-out mice, whereas fibroblasts of wild-type and PGC-1α-deficient animals did not show any difference in RNA polymerase I transcription confirming the tissue-specificity of PGC-1α (Fig. 3c).

To monitor the consequences of PGC-1α association with the rDNA, the co-factor was transfected in HEK cells (3%O_2_) and RNA polymerase I transcriptional activity was analysed with/without resveratrol treatment. Relative 47S transcription was only stimulated in HEK cells after transfection of PGC-1α and addition of resveratrol (Fig. 3d/e) indicating that the expression of endogenous PGC-1α is not sufficient to stimulate RNA polymerase I transcription (PGC-1α mRNA was detected by primers spanning exon 10–11). Endogenous and also transfected PGC-1α mRNA levels are stimulated by addition of resveratrol (Fig. 3e, Supplementary Figure S5), however in the case of the endogenous PGC-1α the critical threshold to enhance RNA polymerase I transcription might not be reached.

Moreover, the induction of rDNA transcription by PGC-1α is at least partially mediated by the rDNA promoter as shown by co-transfection of the minimal-promoter rDNA reporter pHrP_2_BH (Supplementary Figure S5) that was also stimulated by activated PGC-1α.

### PGC-1α stimulates rDNA transcription *in vivo*

**Figure 4:**
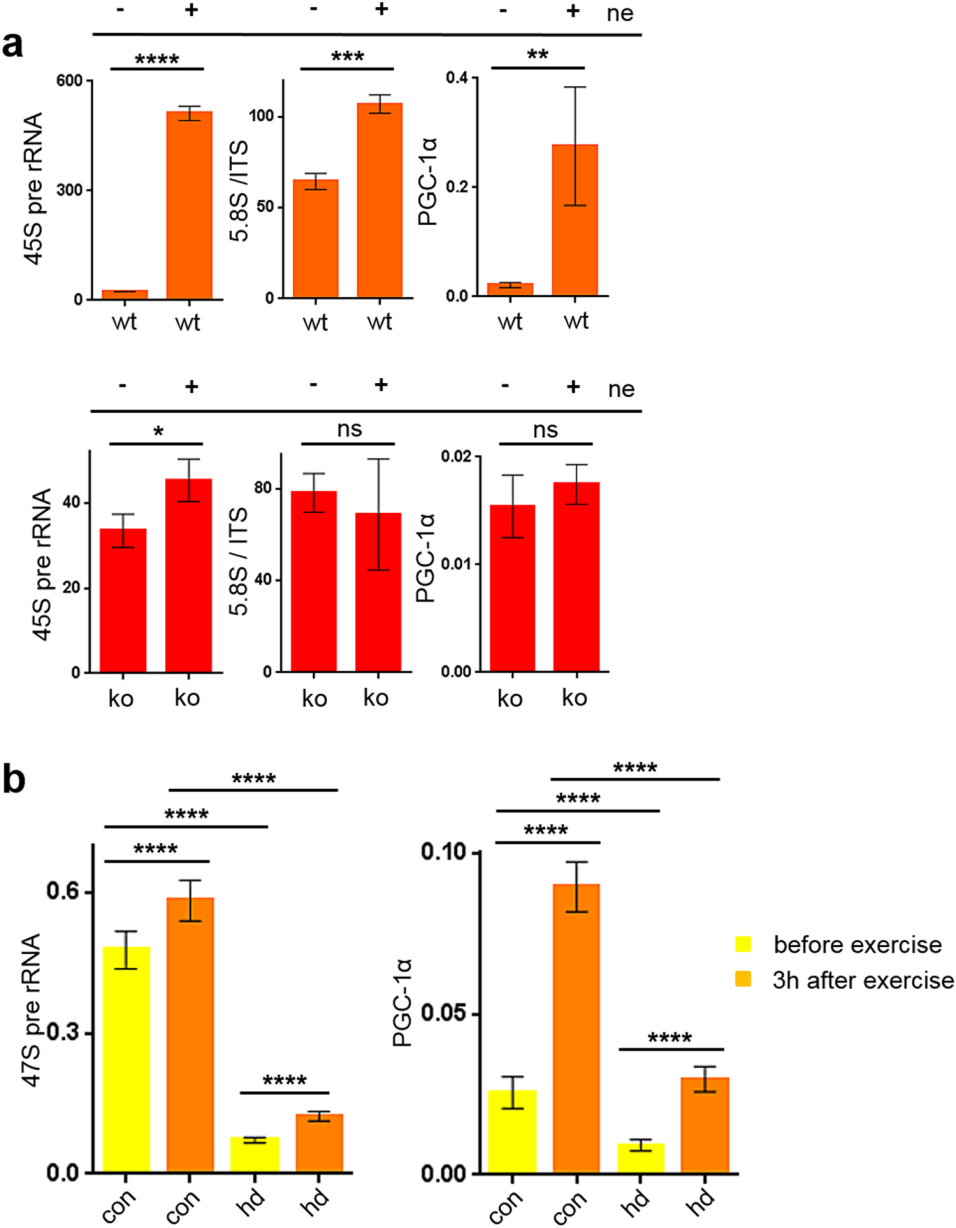
rDNA transcription is severely impaired in PGC-1α ko tissue and in Huntington’s disease. (**4a**) PGC-1α expression induces transcription of the m45S rRNA after treatment with 10 µM norepinephine for 3h. Primary cells from brown adipose tissues of WT and PGC-1α-KO, treated with/without norepinephrine. Samples used in the qPCR are normalized to mRPL13 (mean of the samples is subtracted from the mean of the endogenous control mRPL13). N = 5; *p = 0.0135, **p = 0.0011, ***p < 0.0004, ****p < 0.0001; t-test two tailed; column: mean ± SD. (**4b**) Analysis of rDNA transcription and PGC-1α expression in muscle biopsies of healthy humans and carriers of the Huntington disease mutation is increased 3 h after exercise. Basal and induced values are at much lower levels in the HD patients. Muscle fine-needle biopsies (M. quadriceps femoris) of healthy humans (n = 21) and mutation carriers of Huntington’s disease (n = 7) before and 3 hours after exercise for 30 minutes on a bicycle ergometer. qPCR of cDNA; samples are normalized to hRPL13 (mean of the samples is subtracted from the mean of the endogenous control hRPL13). ****p < 0.0001; column: mean ± SD.

Norepinephrine is a physiological inducer of PGC-1α expression and activation in brown adipose tissue^22^. QPCR analysis of RNA polymerase I transcriptional activity 3h after treatment with norepinephrine revealed a strong activation of Pol I transcription initiation (45S) and elongation (5.8S/ITS) in BAT cells of wild-type, but not in primary cells from KO animals. The basal, unstimulated levels of RNA polymerase I transcription initiation (45S pre-rRNA) are comparable in wildtype and PGC-1α knock-out cells, whereas norepinephrine stimulated in dependence of PGC-1α Pol I initiation up to 100-fold (Fig. 4a). This is accompanied by association of PGC-1α with the rDNA promoter (Figure S6). Importantly, the anti-cancer agent CX5461, which is currently in phase I/II trials (NCT02719977) and therapeutically targets rDNA transcription^23^, inhibits resveratrol induced expression of the PGC-1α target gene complex V (Figure S6). However, in higher dose CX5461 also induces DNA damage^24^.

PGC-1α dysregulation in the CNS and peripheral tissues is a hallmark of Huntington’s disease, an autosomal dominant adult-onset neurodegenerative disease often associated with severe sarcopenia in advanced stages^10, 18^. PGC-1α expression and activity in muscle is induced by exercise^25^ thus we speculated that RNA polymerase I transcriptional activity should also be activated by physical exercise. 21 untrained healthy persons of age 18–65 performed a 30 minutes exercise ergometer training and muscle biopsies were taken before and 3h after exercise. Additionally, we performed muscle biopsies before and 3h after exercise in 7 carriers of the Huntington’s disease mutation (Supplementary Table 1). Total RNA was harvested and PGC-1α expression and RNA polymerase I activity were analysed. As depicted in Fig. 4b, exercise induced PGC-1α mRNA expression and stimulated RNA polymerase I transcription in healthy donors. Although we detected an increase PGC-1α expression and 47S rRNA transcription after exercise in patients with Huntington’s disease, the overall transcriptional activity of RNA polymerase I in muscle tissues was severely compromised. The initiation rate of rDNA transcription was found to be reduced almost 6-fold in gene carriers of Huntington’s disease indicating that rDNA transcription is critically dependent on PGC-1α. These results extend the hypothesis, that PGC-1α regulates rDNA transcription under different stress conditions to the observation that even basal levels of rDNA transcription are affected by a possible dysregulation of PGC-1α in Huntington’s disease.

## Discussion

PGC-1α is expressed in tissues of high metabolic activity like brain, muscle, heart and liver and regulates the energy supply by induction of mitochondrial biogenesis depending on the changing energy demand of the cells. Moreover, ribosomal biogenesis is a highly ATP consuming process so that both, mitochondrial and ribosomal biogenesis seem to be dependent on each other. We hypothesized that PGC-1α as a key transcriptional regulator could not only represent a master regulator of ATP production but also influence ribosomal biogenesis.

First, we showed the association of PGC-1α with the ribosomal DNA and subsequent induction of ribosomal transcription, depending on various stress factors that are known to stimulate PGC-1α like oxidative stress, resveratrol, norepinephrine and exercise. As association of PGC-1α with the rDNA at normal/unstressed conditions requires its activation by resveratrol – a sirtuin mimetic that induces Sirt1 that in turn deacetylates and thus activates PGC-1α – and could be counteracted by nicotinamide, the interaction of PGC-1α with the rDNA might be regulated by a Sirt-1 like activity. It is remarkable that Sirt1 is part of the eNosc complex, known to epigenetically silence the rDNA locus in response to starvation^26^. As our results suggest induction of PGC-1α in a Sirt1 like activity this discrepancy may be due to different stress conditions whereby PGC-1α response to hyperoxia, norepinephrine and resveratrol might differ from that of starvation. PGC-1α and especially truncated NT- PGC-1α expression is activated under hypoxic conditions of 0.5% or 0.2% O_2_^27, 28^, however, we did not observe expression differences between 3% and 21% oxygen (Supplemental Figure 3b).

Second, as a direct binding of PGC-1α to DNA has not been demonstrated yet^29^, we were able to identify UBF as a potential candidate target of PGC-1α. We show a close association of PGC-1α with UBF in co-immunoprecipitation and ChIP-re-ChIP experiments. Moreover, promotor occupancy of RNA polymerase I and in particular UBF are induced by resveratrol treatment and decreased by knock-out of PGC-1α indicating that the latter alters the affinity of UBF to the rDNA promotor and might have a direct stabilizing effect on UBF as shown for the Wnt target Peter Pan^30^. While this constellation allows for the possibility that PGC-1α coactivates UBF, further research is necessary to determine the exact nature of the interaction of these two factors.

Third, we transferred our cell culture based results into the mouse model. Upon ß-adrenergic stimulation, PGC-1α has a well-known stimulatory effect on mitochondrial biogenesis in brown adipose tissue (BAT)^31^. This is accompanied by a strong stimulation of RNA polymerase I transcription that is dependent on PGC-1α as we detected higher transcriptional activity in PGC-1α expressing tissues of WT mice than tissues from PGC-1α KO mice. The physiologic stimulus of PGC-1α activation in BAT is exposure to cold. How rDNA transcription and ribosomal biogenesis contribute to thermogenesis–the main task of BAT–remains to be elucidated.

Although we are still ignorant about the exact role of PGC-1α in Pol I transcription and the mechanisms and consequences of a possible co-regulation of ribosomal and mitochondrial biogenesis, our findings do point towards a novel function for PGC-1α. Given the fundamental significance of ribosomal biogenesis in cell biology, our finding that PGC-1α co-regulates the activity of UBF potentially has broad implications. Ribosomal biogenesis is the key determinant of overall protein synthesis and exquisitely energy demanding^32–34^. A role of the metabolic master regulator PGC-1α in this process thus represents an attractive mode for coordinating energy consumption and energy production in the cell.

A very tangible consequence of this relationship emerges from our examination of this pathway during exercise under physiological and disease conditions. Physical exercise requires energy sources in the form of ATP mostly derived from mitochondria. Physical exercise is an established inducer of PGC-1α in muscle cells^35^. Here we show that physical exercise induced PGC-1α expression in muscle biopsies of healthy persons. This is followed by a significant upregulation of RNA polymerase I transcription in humans – thus validating our insights from model systems. Exercise is a stimulus for remodeling of muscle cells accompanied with an elevated demand for protein synthesis at the ribosomes^36^; for this it is valid to argue that exercise induces ribosomal biogenesis. In a last step, we also link our results to pathological conditions by investigating carriers of the Huntingtin mutation – a disease associated with sarcopenia and cachexia that are not only due to permanent choreatiform movements but also to impairment of metabolic functions^37^. Carriers of the Huntington’s disease mutation showed similar response to exercise with upregulation of PGC-1α expression and increased ribosomal biogenesis in muscle biopsies but with much lower basal and induced levels compared to healthy controls. As mutated Huntingtin is known to repress PGC-1α transcription^10, 18^, ribosomal biogenesis may be critically compromised with impaired capacity of protein synthesis in cells resulting in sarcopenia and cachexia. Moreover a compromised ribosomal biogenesis even in early stages of HD is raising the prospect that this could be used as a diagnostic target to examine sarcopenia and cachexia in Huntington’s disease.

## Methods

### Antibodies

PGC1a mouse monoclonal (4C1.3), Calbiochem/ST1202, raised against aa 1–120 of mPGC-1α (ChIP); PGC-1α rabbit polyclonal, Millipore/AB3242 raised against aa777–797 of mPGC-1α (IF); Nucleolin C23 (F-18) goat polyclonal, Santa Cruz/sc9893; Immunoglobulin G, mouse Santa Cruz/sc-3877; RNA-Polymerase I, Lebedev *et al*. 2008; Anti-FLAG M2, Sigma A2220 (IP); Acetyl-lysine mouse monoclonal, Acris/AM33097PU-S.

### Plasmids

pcDNA-f:PGC1 mouse, FLAG-tag, addgene/1026; pUC18; pHrP_2_BH plasmid containing the minimal human rDNA promoter ahead of a pUC9 sequence and a terminator element (kind gift of I. Grummt).

### Immunofluorescence staining

100.000 HEK or N2A cells were disseminated on polylysine coated coverglasses and incubated in DMEM over night. Cells were fixed with paraformaldehyde for 15 minutes and blocked with BSA 5% for 1h at room temperature. 1. Antibodies (AB) were incubated over night: IgG 1:200, PGC-1α Millipore AB3242 1:200, nucleolin 1:200. Second AB used for immunofluorescence: alexa 555 or 488 1:200 with incubation-time of 45 minutes. Staining of nuclei was performed with DAPI 1:5000.

### Western blotting

The immunoprecipitated proteins were heated in 1x Laemmli buffer for 5 min at 99 °C and then subject to 10% SDS-PAGE. Immunoprecipitated chromatin was heated for 45 min at 99 °C in 1x Laemmli buffer and resolved on a 10% SDS-gel. A semi-dry blotting system (BioRad) was used to transfer the proteins to nitrocellulose (Protran BA-85, GE) and after blocking in 5% skim milk, 0.05% Tween 20 in PBS incubated over night with the indicated antibody.

### Extraction of chromatin from tissues/cells

Tissues were grinded on liquid nitrogen. Tissues and cells were resuspended in PBS and fixed with 1% formaldehyde for 10 minutes to crosslink proteins to DNA. Crosslinking was stopped by addition of 0.125M Glycine for 5 minutes. Next steps were washing with PBS several times and centrifugation of the pellet at 5000 rpm. The pellet was resuspended in cell-lysis buffer and incubated on ice for 10 minutes. After re-centrifugation, the pellet was resuspended in 1000 µl buffer N and 1 µl DTT. Addition of 100 µl 10x micrococcal nuclease buffer, 10 µl 100xBSA and 6 µl micrococcal nuclease were necessary for digest of the DNA that was performed at 37 °C for 40 minutes. Digest was stopped by 0.5M EDTA. After centrifugation, the pellet was resuspended in nuclei-lysis buffer plus 1:50 complete and incubated in ice for 10 minutes. An additional step of sonication was performed to shorten the DNA fragments of approximately 150–900 bp. DNA-fragment size was determined by electrophoresis on a 1% agarose gel with a 1 kb marker.

### Chromatin immunoprecipitation assay (ChIP)

Pre-clearing was performed to remove non-specific background from proteins. 50 µl chromatin was mixed with 200 µl IP dilution buffer, 5 µl complete and 20 µl salmon sperm-DNA/protein A agarose beads and incubated at 4 °C for 30 minutes. Beads were pelleted by centrifugation for 3 minutes at 6000 rpm. 250 µl of the supernatant was incubated with 2 µg of specific antibodies (rabbit/mouse IgG, Pol I, PGC-1α (flagM2/CalbiochemST1202)) over night at 4 °C with rotation. 20 µl protein A agarose beads/ss DNA were added for 1h to capture the antibody. For washing, the beads were resuspended in 1000 µl low salt buffer and transferred into new tubes to reduce background signals from chromatin absorption to the plastic. The samples were incubated at 4 °C for 5 minutes with rotation. After centrifugation, the pellet was resuspended with 1000 µl high salt buffer and incubated at 4 °C for 5 minutes. After centrifugation, the pellet was washed with 1000 µl LiCl buffer and incubated at 4 °C for 5 minutes. After re-centrifugation, the pellet was washed twice with 1000 µl TE buffer and incubated at 4 °C for 5 minutes. For elution, the pellet was mixed with 150 µl µChIP elution buffer and 2 µl proteinase K and incubated at 65 °C for 2 h. For DNA purification, the supernatant was treated according to the manufacturer’s protocol (QIAquick Nucleotide Removal Kit).

### ChIP-re-ChIP

Proceeding was according to the protocol for ChIP until washing with TE buffer. 25 µl TE buffer/10mM DTT were added to the beads and shaken at 37 °C for 30 minutes. After centrifugation at 3000 rpm for 2 minutes, 25 µl of the supernatant were removed into a separate tube. The beads were incubated with 475 µl IP dilution buffer for 5 minutes at 4 °C with rotation. After centrifugation, 475 µl of the supernatant were mixed with the 25 µl taken before and second antibody for the Re-ChIP was added. Incubation was performed over night. Washing, elution and DNA purification steps were done as described for ChIP. For ChIP-analysis, we performed the following steps: Samples were normalized to native chromatin as positive control, named input. For this, mean of technical triplicates of the sample were subtracted from the mean of technical triplicates of input followed by raising the power to 2^(Cycle threshold Input – Cycle threshold Sample)^ and multiplied with 100 to get %input.

### Immunoprecipitation

300 µg HEK lysate, transfected with flag-PGC-1α were used per sample and incubated with 3–4 µg flag-(M2)antibody for 1,5h at 4 °C by rotation. 25 µl protein A sepharose was added per sample and incubated 1h at 4 °C by rotation. After centrifugation with 6000 rpm for 2.5 minutes, supernatant was discarded and beads were washed thrice with 200 µl AM100 buffer. After adding of 20 µl AM-100 buffer, 10 µl Laemmli and 5 µl β-Mercaptoethanol, proteins were denaturated at 99 °C for 10 minutes. Samples were subject to SDS-PAGE, blotted for 1 h and blocked in 5% milk containing 10% Tween for 1 h. Antibodies were incubated over night at 4 °C using a dilution of 1:500. After washing with PBS the membrane was incubated with the second antibody (dilution 1:10000) for 1 h. The membrane was developed using chemiluminescence.

### Isolation of genomic DNA from tissues/cells

Tissues were grinded on liquid nitrogen. A mix of TE-buffer (320 µl), Solution A (20 µl), Solution B (10 µl) (invitrogen) and proteinase K (8 µl) was added to tissues and to washed cell plates, respectively. Digest was performed for 30 minutes at 60 °C. 300 µl solution A and 120 µl solution B were added and the sample was vortexed until the solution was viscous. 750 µl chloroform was added to the sample and vortexed until the solution was homogenous. After centrifugation at 4 °C for 10minutes, the upper aqueous phase was transferred to a new tube. 1ml of 100% ethanol (−20 °C) was added and the sample was incubated in ice for 30 minutes. After centrifugation, the pellet was washed with 500 µl 80% ethanol (−20 °C). After centrifugation, the pellet was air-dried for 5 minutes and resuspended in TE-buffer containing RNAse to a final concentration of 40 µg/ml. The sample was incubated at 37 °C for 30 minutes. Concentration was measured using nanodrop.

### RNA extraction

RNA extraction was performed using the Qiagen-kit RNeasy mini kit according to the manufacturer’s specification. Concentrations were measured using nanodrop.

### Reverse transcription

1 µg RNA was mixed with 1 µl N6 primer and water to 5 µl and incubated for 5 minutes at 70 °C. Afterwards, 20 µl of the following mix was added: 0.5 µl dNTP, 13 µl aqua bidest, 5 µl 5xM-MLV buffer, 0.5 µl RNAsin and 1 µl M-MLV. Incubation was performed at 37 °C for 1h.

### Calcium-phosphate transfection

Cells were split into 15cm tissue culture dishes the day before transfection. 100 µl 2.5M CaCl2, 900 µl sterile water, 30µg plamid-DNA PGC-1α and 1µg pEGFP were mixed. The solution was added dropwise to 1000 µl HeBS buffer (pH 7.03) under vortexing and then incubated for 20 minutes at room temperature. The precipitate was distributed over the tissue culture dish. After 48h, cells were lysed for further protein purification.

### Statistical analysis of mean signal intensities (confocal microscopy)

Mean signal intensities of DAPI, PGC-1α, and Nucleolin in the nucleolus of HEK and N2A cell lines were compared by Mann-Whitney-Wilcoxon test using R statistical package. Overlaps between distributions of mean intensities were evaluated by density plots.

### Animals

Genotype of the used animals: B6.129-Ppargc1a^tm1Brsp^ /J, strain B6 from The Jackson Laboratory: https://www.jax.org/strain/008597. PGC-1α exon 3–5 are deleted and detected as described in the link above. For tissue analysis 90 days old female mice were sacrificed. BAT for *in vitro* experiments was explanted from animals postpartal day 0–2. Generation N10 + N1F3/4.

### Muscle biopsies after exercise

In a first appointment a physical examination and an endurance test to evaluate the maximal performance on a bicycle ergometer was performed. In a second appointment a fine-needle biopsy from the quadriceps femoris was taken. A third term included an exercise of 60% of maximal endurance for 30 minutes on the bicycle ergometer, then a fine-needle muscle biopsy was taken after 3h recovery.

### Study approval

All methods were carried out in accordance with relevant guidelines and regulations. Mice were bred under approved conditions of the animal facility of Ulm University and the Regierungspraesidium Tuebingen (Reg. C. 0177). The work with human subjects in this study was carried out in accordance with the ethical committee of Ulm University (Nr. 78/14) in compliance with the guidelines of the federal government of Germany and the declaration of Helsinki. Muscle samples were taken from the middle of the belly of the right musculus vastus lateralis by a fine-needle biopsy technique using a 14G biopsy needle and a 13G puncture cannula (Pflugbeil, Zomeding, Germany) after disinfection and local anesthesia. Muscle tissue of about 4–5 mg were immediately transferred to the experiments within 45 min after biopsy. All of the human participants gave written informed consent to the study.

### Data availability

The datasets generated during and/or analysed during the current study are available from the corresponding author on reasonable request.

## Electronic supplementary material

Supplement
