# Supplementary material for: Ribosomal transcription is regulated by PGC-1alpha and disturbed in Huntington’s disease
Source: Sci Rep. 2017 Aug 17;7:8513. doi: 10.1038/s41598-017-09148-7 (PMC5561056; doi:10.1038/s41598-017-09148-7)

Ribosomal transcription is regulated by PGC-1alpha and disturbed in Huntington´s disease

Sarah Jesse[1], Hanna Bayer[2], Marius C. Alupei[3], Martina Zügel[4], Medhanie Mulaw[5], Francesca Tuorto[6], Silke Malmsheimer[3], Karmveer Singh[3], Jürgen Steinacker[4], Uwe Schumann[4], Albert C. Ludolph[1], Karin Scharffetter-Kochanek[3], Anke Witting[2], Patrick Weydt[1,*] and Sebastian Iben[3,*]

Supplementary Information

Figure S1, related to Figure 1A

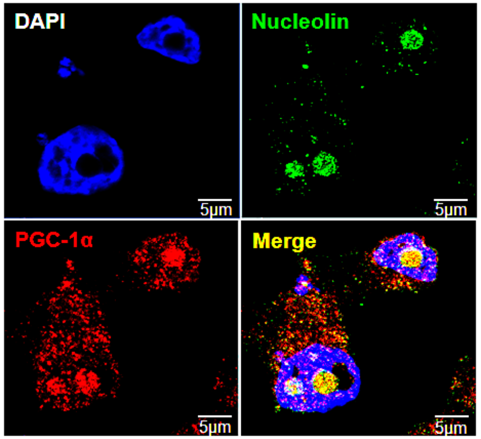

Immunocytochemical stainings in untreated N2A cells kept under 21% pO_2 show co-localization of PGC-1α and nucleolin in the merge. Confocal microscopy.

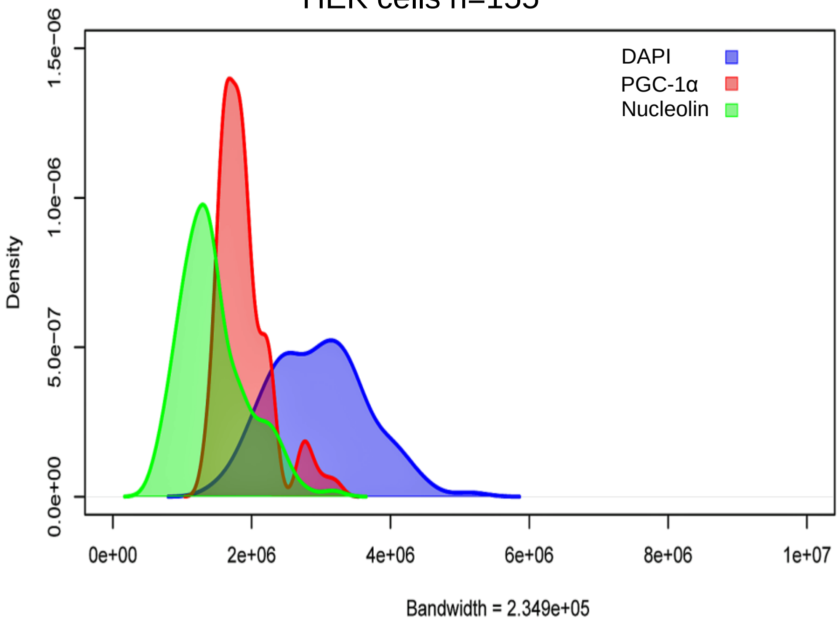

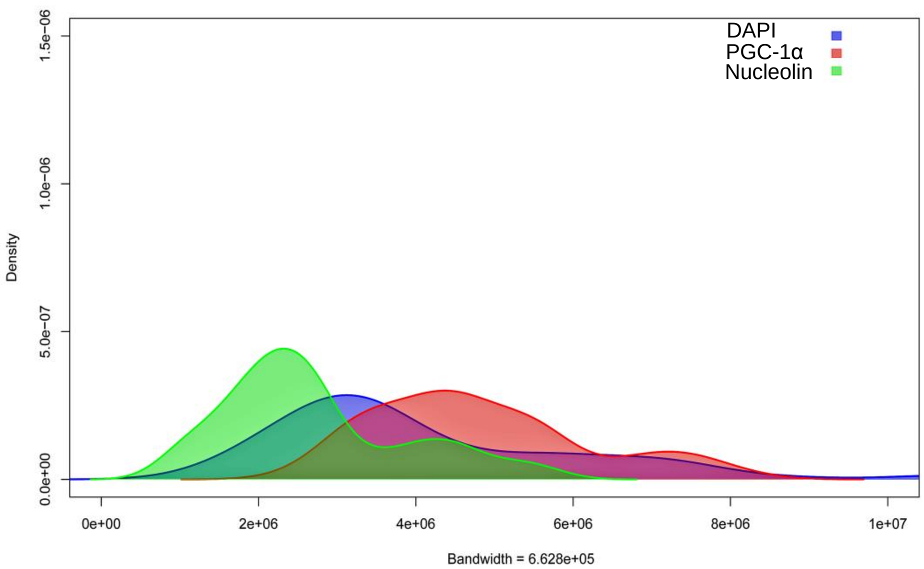

Figure S2, related to Figure 1A. Density plots show distribution and overlap between mean signal intensities of DAPI (blue), PGC-1α (red) and Nucleolin (green) in the nucleolus of HEK and N2A cell lines. Stronger overlaps indicate higher similarity in the distribution and levels of mean signal intensities between markers. Both cell lines show a strong overlap and no significant difference in the mean intensities of PGC-1α (red) and Nucleolin (green) in the nucleolus.

Figure S3, related to Figure 1A

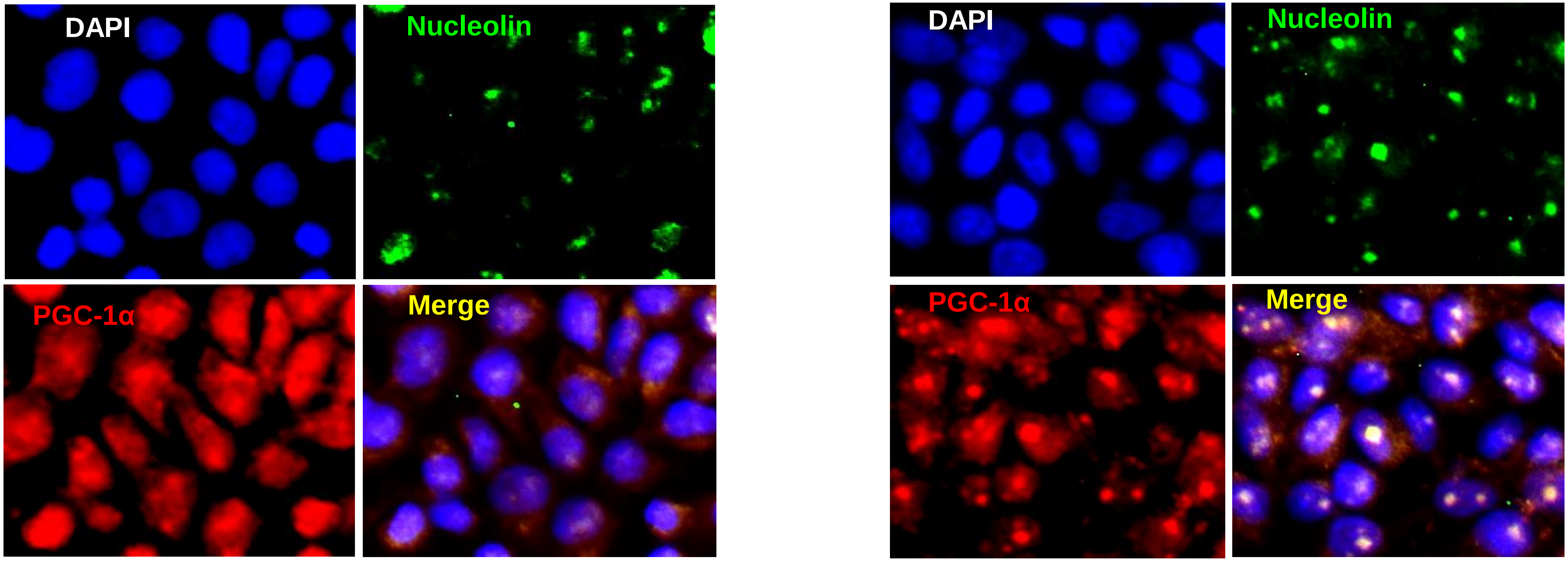

HEK 3% pO_2 **HEK 3% pO_2 + resveratrol**

Immunofluorescence of HEK cells kept under 3% pO_2 with/without resveratrol treatment shows condensed localization of PGC-1α in the nucleoli only after stimulation with resveratrol.

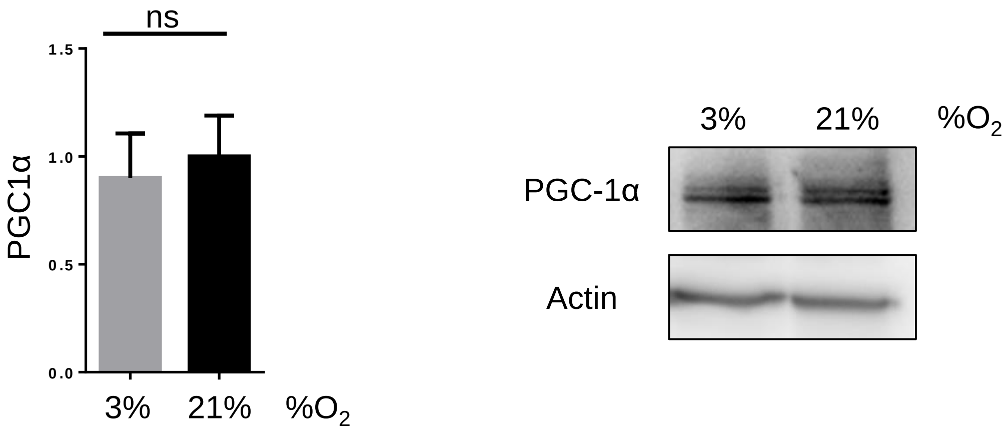

QPCR/Western blot analysis of PGC-1a abundance in HEK cells kept under different O_2 conditions

Figure S4, related to Figure 1B

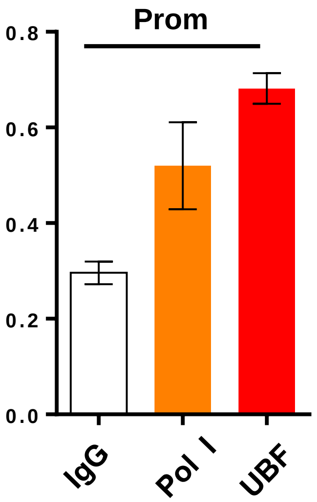

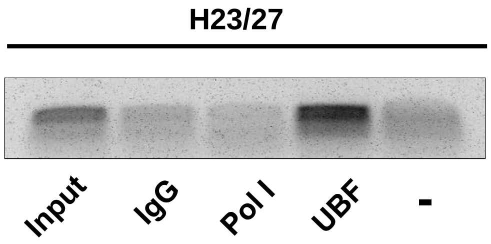

UBF binds strongly to the sequences amplified by H23/27 (ChIP)

Figure S5, related to Figure 3D

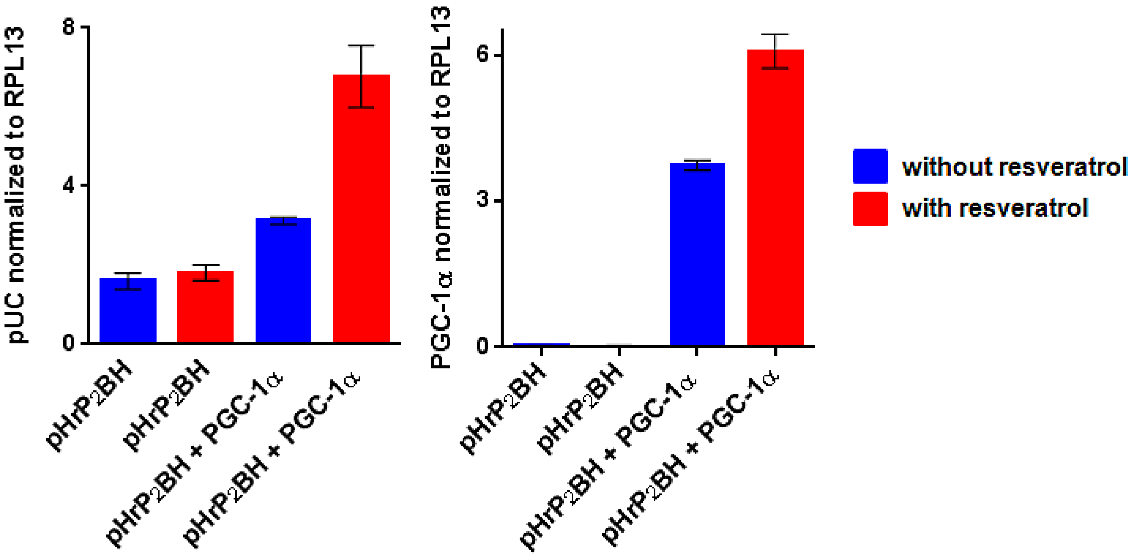

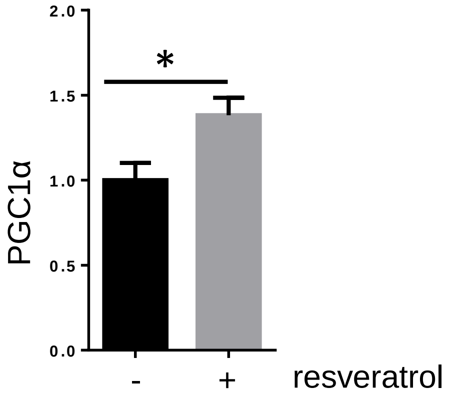

rDNA transcription by PGC-1α is at least partially mediated by the rDNA promoter as shown by co-transfection of the rDNA reporter $pHrP_2BH$ containing the human rDNA core promotor and a short coding region, followed by pUC-sequences.

Reveratrol stimulates basal PGC-1a Expression in HEK cells

Figure S6

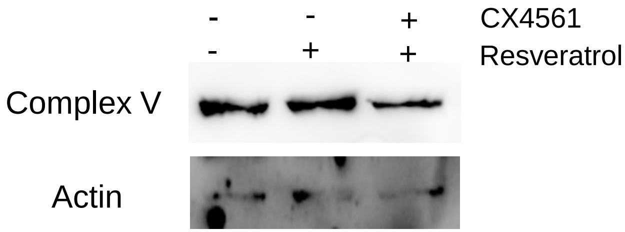

The PGC-1alpha target complex V expression is stimulated in the presence of resveratrol.
This stimulation is blunted in the presence of the RNA polymerase I inhibitor CX5461.
HEK cells were transfected with PGC-1alpha, and treated for 96h with the indicated drugs.
Lysates (20ug) were analysed by Western blot with the indicated antibodies.

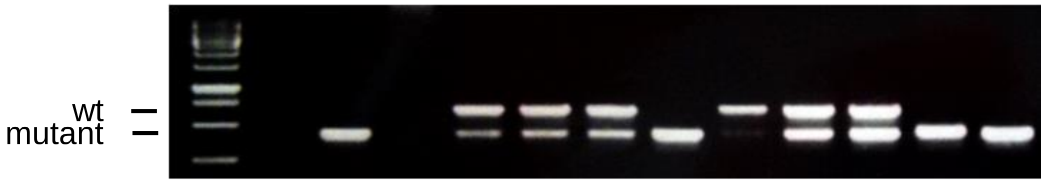

Agarose-gelelectrophoresis of a typical genotyping experiment with DNA of wt, heterozygous and ko animals.
(https://www2.jax.org/protocolsdb/f?p=116:2:0::NO:2:P2_MASTER_PROTOCOL_ID,P2_JRS_CODE:5061,008597)

Figure S7, related to Figure 4A

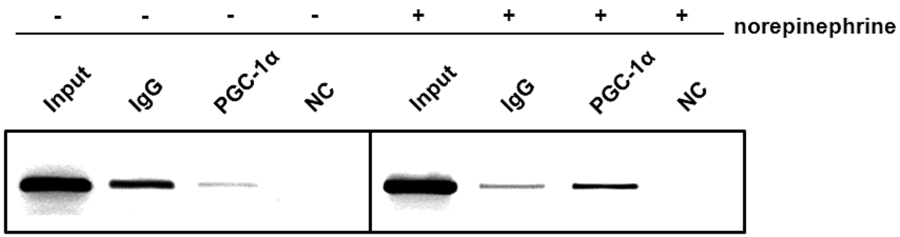

ChIP using HEK cells grown under 3% pO_2 with/without norepinephrine treatment shows binding of PGC-1α to the rDNA promotor only after activation with norepinephrine. Semi-quantitative PCR using primers for rDNA promotor.

Figure S7

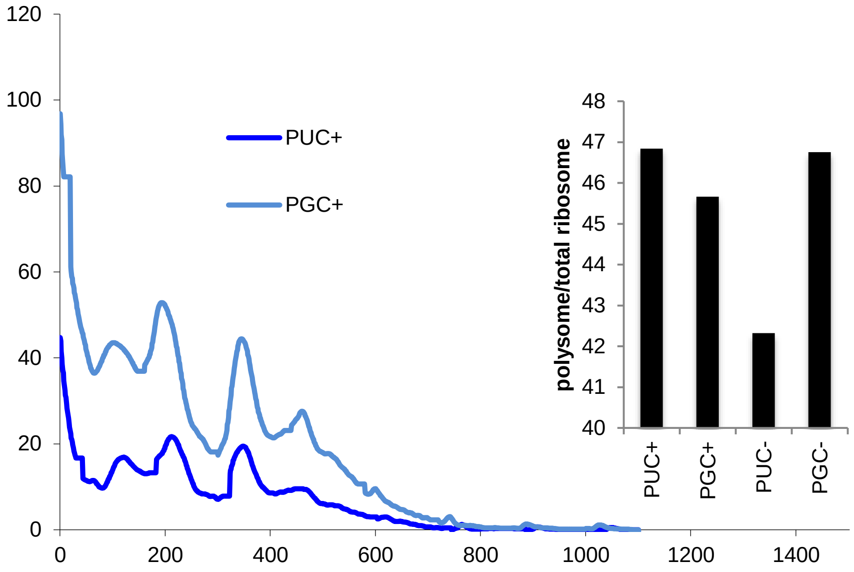

Polysomal profiles of pUC or PGC1α-transfected HEK cells, treated with resveratrol (+). The right panel shows the quantification of the polysomal fraction, normalized to the total number of ribosomes.

HEK cells were transfected with pUC or PGC1α and treated for 24 h with/without resveratrol, treated for 5 min with cycloheximide (200 ng/ml), washed with PBS containing cycloheximide (200 ng/ml), and lysed (20 mM Tris-HCl, pH 7.4, 5 mM MgCl2, 150 mM NaCl, 1% Triton X-100, 0.1%, 100 µg/ml cycloheximide, 1×Complete Protease Inhibitors (Roche)). After 10 min centrifugation at 10000 r.p.m. at 4 °C, the lysates were applied to a linear 17.5-50% sucrose gradients in 15 mM Tris-HCl, pH 8.0, 15 mM MgCl2, 300 mM NaCl. Gradients were centrifuged at 36,000 r.p.m. for 2.5 h at 4 °C in a Beckman SW 60 rotor. The polysomal profiles were obtained by fractionation (Brandel GradFrac) and continuously recorded by monitoring the absorbance at 254 nm and further calculated by dividing the area under the polysomal part of the curve to the area below the entire curve.

Figure S8 Western blot raw data

related to figure 2e

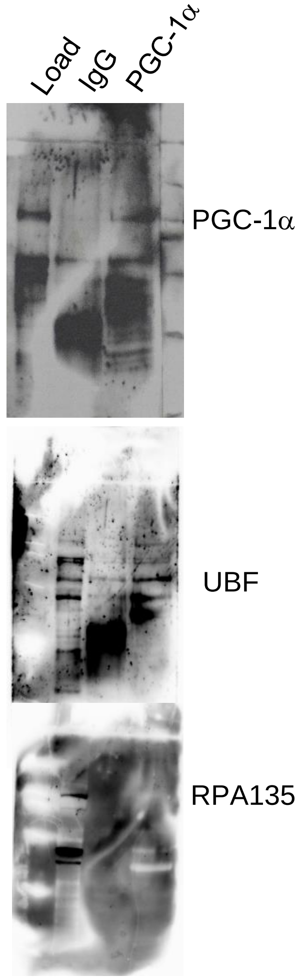

related to figure 2b

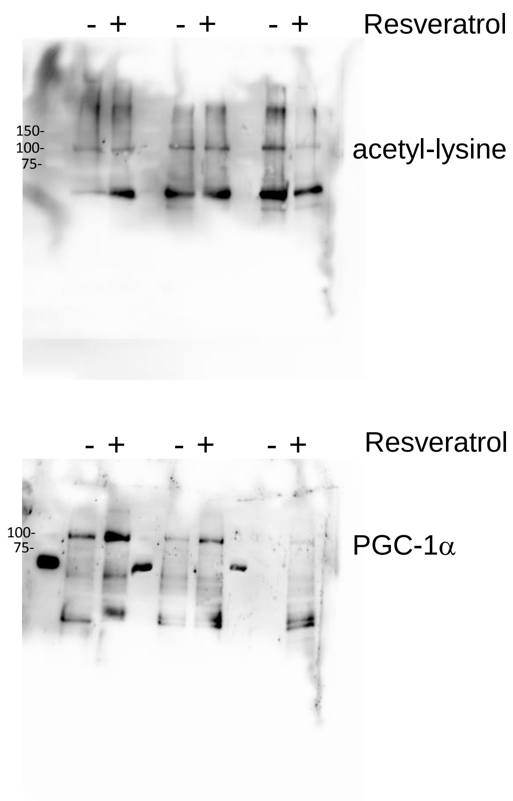

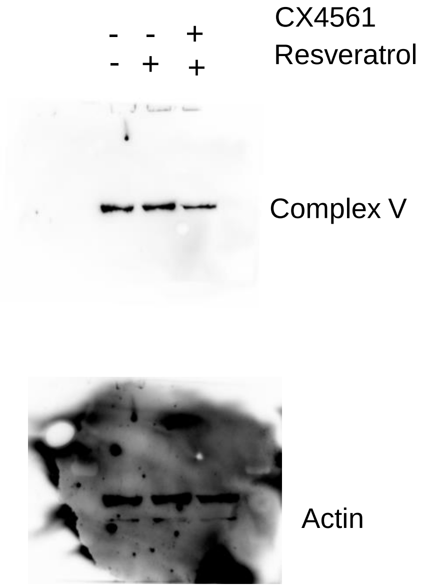

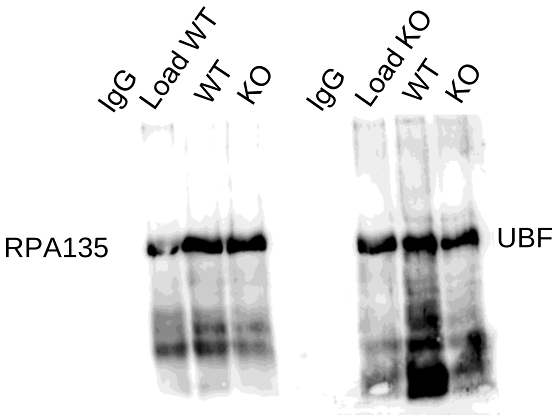

related to figure 3a

Supplementary Table 1: Primer sequences, plasmids used

Primer	Sequence
hPGC1α for	TGAGAGGGCCAAGCAAAG
hPGC1α rev	ATAAATCACACGGCGCTCTT
hIGS for	CCACAGGTCGAGGCTTCGGT
hIGS rev	GGCGCTCTTCTGCGTCTCACT
H23/27 for	CCTTCCACGAGAGTGAGAAGC
H23/27 rev	TCGACCTCCCGAAATCGTACA
HrChIP for	CTGCGATGGTGGCGTTTTTG
HrChIP rev	ACAGCGTGTCAGCAATAACC
H4 for	GACGACCCATTCGAACGTCT
H4 rev	CTCTCCGGAATCGAACCCTG
H8 for	AGTCGGGTTGCTTGGGAATGC
H8 rev	CCCTTACGGTACTTGTTGACT
h47S rRNA for	TGTCAGGCGTTCTCGTCT
h47S rRNA rev	AGCACGACGTCACCACAT
m45S rRNA for	CGTGTAAGACATTCCTATCTCG
m45S rRNA rev	GCCCGCTGGCAGAACGAGAAG
hRPL13 for	CGGACCGTGCGAGGTAT
hRPL 13 rev	CACCATCCGCTTTTTCTTGTC
mRPL13 for	ACCTCCTCCTTTCCCAGGCGG
mRPL13 rev	CGCCAGGCGGCCAAGAAGAT
LMNA for	GGGAAAGAATGGGAGGAGAG
LMNA rev	ATGCCTGCAATTACCAGGAG
PCK1 for	GTGCATCCTTCCCATGAACT
PCK1 rev	GCCCTCAACCAACCATAGTG
IRS1 for	ACACCCATTGAACCACCCTA
IRS1 rev	CGTTTGTTTGTGGGGAGACT
m5.8S for	ACACATTGATCATCGACAC
m5.8S rev	TGGCCCTGCGAGCAAACTCCCA

Plasmid	Company/number
pcDNA-f:PGC1 mouse, FLAG-tag	addgene/1026
pcDNA4 PGC1 human, Myc-, His-tag	addgene/10974
pUC 18	/

Supplementary Table 2: Huntingtons disease carriers:

	Age	sex	BMI	cag short	cag long
HD1	53	m	21,5	23	43
HD2	32	w	21,5	22	47
HD3	21	m	25,35	26	47
HD4	52	m	23,07	17	41
HD5	64	m	24,83	15	42
HD6	34	m	29,63	20	43
HD7	60	w	28,84	17	40

Graphic of primers used, related to experimental procedures

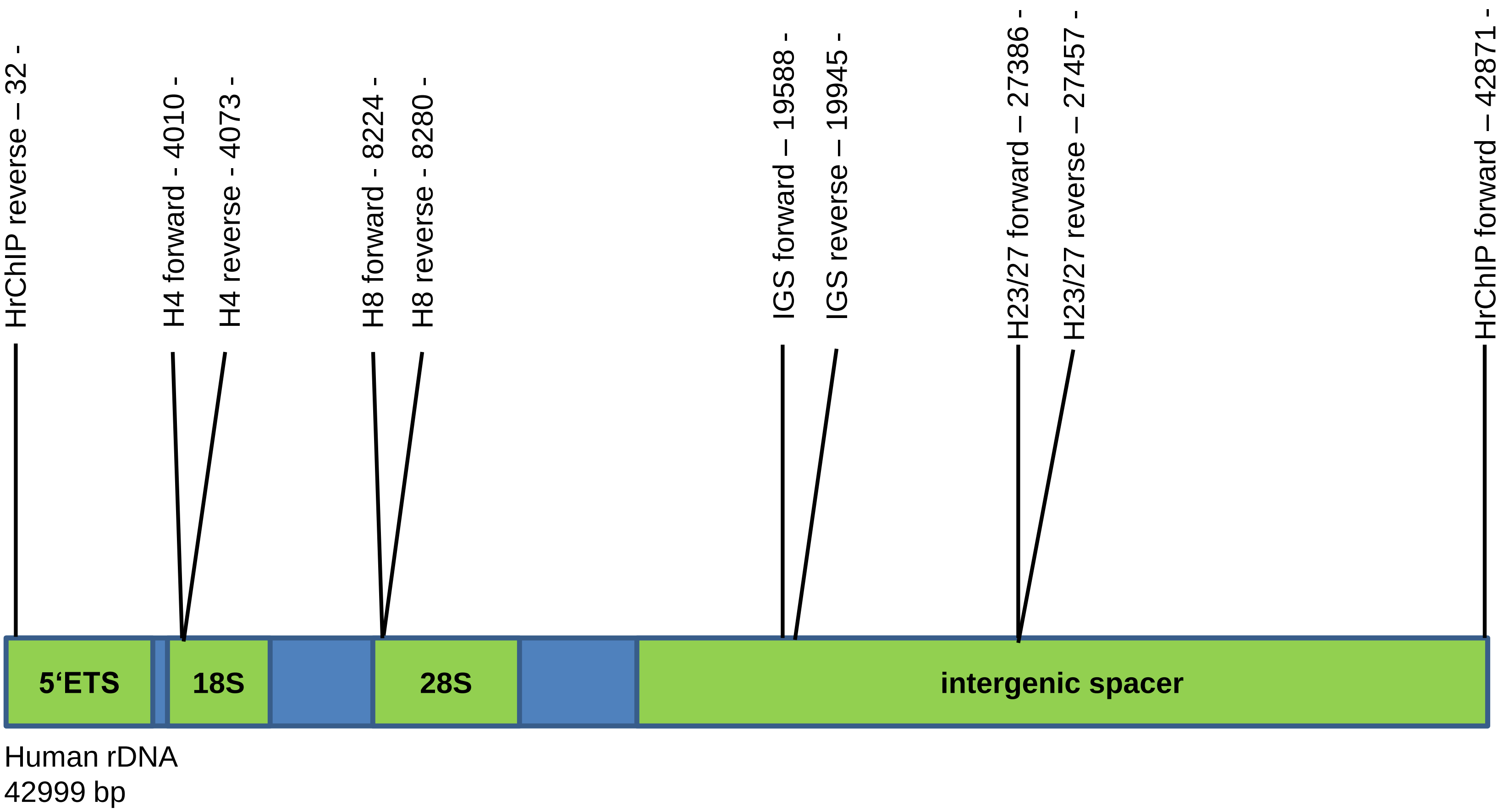

Supplement: Supplementary file 1 — Supplement [file 41598_2017_9148_MOESM1_ESM.pdf]
